# Association between the luteinizing hormone/chorionic gonadotropin receptor (*LHCGR*) rs4073366 polymorphism and ovarian hyperstimulation syndrome during controlled ovarian hyperstimulation

**DOI:** 10.1186/1477-7827-11-71

**Published:** 2013-07-25

**Authors:** Travis J O’Brien, Mariah M Kalmin, Arthur F Harralson, Adam M Clark, Ian Gindoff, Samuel J Simmens, David Frankfurter, Paul Gindoff

**Affiliations:** 1 Department of Pharmacology and Physiology, The George Washington University, Washington, DC, USA; 2 Department of Epidemiology and Biostatistics, The George Washington University, Washington, DC, USA; 3 Department of Pharmacogenomics, Bernard J. Dunn School of Pharmacy, Shenandoah University, Ashburn, VA, USA; 4 Department of Obstetrics and Gynecology, The George Washington University, Washington, DC, USA

**Keywords:** Ovarian stimulation, Ovarian hyperstimulation syndrome, OHSS, LHCGR, LHR, Polymorphism

## Abstract

**Background:**

The aim of this study was to determine the relationship between a purported luteinizing hormone/chorionic gonadotropin (*LHCGR*) high function polymorphism (rs4539842/*ins*LQ) and outcome to controlled ovarian hyperstimulation (COH).

**Methods:**

This was a prospective study of 172 patients undergoing COH at the Fertility and IVF Center at GWU. DNA was isolated from blood samples and a region encompassing the *ins*LQ polymorphism was sequenced. We also investigated a polymorphism (rs4073366 G > C) that was 142 bp from *ins*LQ. The association of the *ins*LQ and rs4073366 alleles and outcome to COH (number of mature follicles, estradiol level on day of human chorionic gonadotropin (hCG) administration, the number of eggs retrieved and ovarian hyperstimulation syndrome (OHSS)) was determined.

**Results:**

Increasing age and higher day 3 (basal) FSH levels were significantly associated with poorer response to COH. We found that both *ins*LQ and rs4073366 were in linkage disequilibrium (LD) and no patients were homozygous for both recessive alleles (*ins*LQ/*ins*LQ; C/C). The *ins*LQ variant was not significantly associated with any of the main outcomes to COH. Carrier status for the rs4073366 C variant was associated (*P* = 0.033) with an increased risk (OR 2.95, 95% CI = 1.09-7.96) of developing OHSS.

**Conclusions:**

While age and day 3 FSH levels were predictive of outcome, we found no association between *ins*LQ and patient response to COH. Interestingly, rs4073366 C variant carrier status was associated with OHSS risk. To the best of our knowledge, this is the first report suggesting that *LHCGR* genetic variation might function in patient risk for OHSS.

## Background

Controlled ovulation hyperstimulation (COH) is the cornerstone of assisted reproduction. The use of exogenous gonadotropins subverts the natural process of single follicular dominance and allows for the recruitment and maturation of multiple ova during the ovarian cycle. Although this technique has vastly enhanced the potential for *in vitro* fertilization (IVF) success, individual patients still have disparate responses. Various phenotypic predictors of ovarian responsiveness (i.e. ovarian reserve testing (ORT)) [1-4] have been used to titrate doses of fertility medications. Despite such predictors, gonadotropin dosing remains somewhat empiric and thus, patients risk under or over responding (ovarian hyperstimulation syndrome (OHSS)) to these drugs. Pharmacogenetic biomarkers offer promise for aiding in the *a priori* determination of patient response to COH [5] and minimizing complications. To date, most work has focused on common variant alleles of follicle stimulating hormone receptor (*FSHR*) [6-17], estrogen receptor (*ESR*) [6-8,18-20] and aromatase (*CYP19A1*) [6,21] genes as well as several other genetic loci [8,10,22,23] which have offered some promising predictive biomarkers for COH outcome.

The luteinizing hormone receptor (LHR) protein is a G protein-coupled hormone receptor (GPCR) [24] and is expressed in numerous tissues including the gonads [25,26], uterus [26-28], fallopian tubes [29], placenta and fetus [30]. Both LH and human chorionic gonadotropin (hCG) are endogenous ligands for LHR [31] with the latter also employed during COH. Prior to ovulation, FSH and estradiol both increase pituitary production of LH and induce LHR expression in the ovaries where LHR functions in promoting follicular maturation, lutenization and ovulation [32]. During assisted reproduction, it is believed that hCG administration (i.e. hCG trigger) activates LHR-mediated signaling. The pharmacological use of hCG for COH has been implicated in the increased ovarian vascular permeability associated with OHSS, an iatrogenic complication of COH [33-36].

LHR is encoded by the luteinizing hormone/chorionic gonadotropin receptor (*LHCGR*) gene (~69 kb) located on chromosome 2p21 [37-39]. *LHCGR* harbors at least 300 known polymorphisms [40-42] some of which having a significant impact on sexual development and fertility [43-51]. Recently, a 6 base-pair (CTCCAG) insertion in exon 1 (rs4539842; *ins*LQ) has been reported that results in the addition of two amino acids (Leu-Gln) in the signal peptide region of the receptor [42,52-54]. The allelic frequency of the *ins*LQ polymorphism is approximately 0.3 in individuals of Caucasian and African descent [54,55]. Structurally, *ins*LQ impacts LHR by potentially altering protein folding, trafficking and membrane insertion. Functionally, the *ins*LQ variant LHR protein displays higher activity in cell culture potentially due to improved trafficking and increased cell surface expression, but unrelated to alterations in hCG binding [52]. Breast cancer patients carrying the *ins*LQ allele exhibit shorter disease-free survival than non-carriers [52,53]. In addition, *LHCGR* resides near a potential susceptibility locus for polycystic ovary syndrome (PCOS), which is characterized by anovulation/oligo-ovulation and elevated androgen levels [56]. In a small case/control study (n = 72), *ins*LQ was detected at a higher level (40.5%) in patients who experienced ovarian hyperstimulation syndrome (OHSS) compared to controls (27.5%), but this did not achieve statistical significance potentially due to a small sample size [57].

Patient response to COH is multi-factorial in nature. To date, there is no reliable test or algorithm that combines both genetic and clinical factors for predicting patient response to fertility therapy. As a result, the focus of this ongoing work is to identify genetic factors that are predictive of clinical response to COH. The *ins*LQ allele appears to result in an LHR protein with increased *in vitro* activity; it is therefore possible that this polymorphism could significantly impact patient response to COH. The objective of this study was to investigate the relationship between the *LHCGR ins*LQ and clinical response to COH. Interestingly, the results suggest that *ins*LQ has little impact on COH outcome. However, carriers of a single nucleotide polymorphism (rs4073366 G > C) nearby *ins*LQ exhibited an increased risk of developing OHSS.

## Methods

This study was approved by George Washington University Institutional Review Board. Written informed consent was obtained from the participants of this study. The study was open to all adult (>18 years of age), female patients seeking treatment at the GW Fertility and IVF Center at GWU Medical Center. One hundred seventy-two patients were recruited into the study from 2010–2011. All IVF patients being monitored while taking injectable gonadotropins were invited to participate. Prior to beginning treatment, all patients had undergone an evaluation that included ovarian reserve testing, semen analysis (male partner), uterine cavity study and thyroid screening. Controlled ovarian stimulation was accomplished with either luteal down regulation using a GnRH agonist (leuprolide acetate; TAP Pharmaceuticals) followed by recombinant FSH (Follistim; Merck & Co; or Gonal-F, Serono) administration or with recombinant FSH (Follistim; Merck & Co; or Gonal-F, Serono) administration in combination with a GnRH antagonist (Antagon; Merck & Co). The initial FSH dose was dependent upon patient age and basal ovarian reserve testing. After initial follicular monitoring (serum estradiol and transvaginal ultrasound assessments), FSH dosing was titrated based upon the ovarian response. hCG trigger was withheld for levels E2 over 4000 pg/ml thus minimizing risk for OHSS. Both control and OHSS groups had similar risk factors including those identified at time of hCG trigger. Ovarian response to gonadotropin was observed. The primary endpoint of the study was the estradiol level on day of hCG injection. Secondary clinical endpoints included number of ovarian follicles counted on day of hCG number of eggs retrieved and the incidence of OHSS. Ovarian hyperstimulation syndrome was defined clinically based on the criteria established by Navot [58,59].

Approximately 5 mL of blood was collected for genetic analysis at the GW IVF Center. Total genomic DNA was extracted from 200 μl EDTA anti-coagulated venous blood using a QiaCube automated instrument with the QIAamp DNA Blood Mini Kit (Qiagen, Valencia, CA). The samples were frozen at −80°C until the time of genotyping. A 291 base-pair target region encompassing (rs4539842/*ins*LQ and rs4073366 was amplified using 10 pmol/μl forward (5’-CACTCAGAGGCCGTCCAAG-3’) and reverse (5’-GGAGGGAAGGTGGCATAGAG-3’) primers [42]. PCR was performed in a reaction volume of 50 μl, including 10.0 μl of 5X Reaction Buffer, 0.5 μl Go*Taq* L nucleotide mix, 6 μl MgCl_2_ DNA Polymerase (Promega Corp, Madison, WI), 3.75 μl of H_2_O, and 10.0 μl of genomic DNA. The PCR cycling conditions were as follows: 1 cycle of 95°C for 2 minutes, followed by 32 cycles of 95°C for 30 seconds, 56°C for 30 seconds and 72°C for 30 seconds and a 7 min final extension at 72°C. PCR products were purified (GeneClean Turbo, MP Biomedicals, Solon, OH) and 20–40 ng/μl of samples were sequenced using BigDye Terminator technology (Life Technologies, Grand Island, NY) by Eurofins MWG Operon (Huntsville, AL). Multiple sequence alignments were carried out using ChromasPro software (Technelysium Pty Ltd).

Patient differences in mean outcomes (estradiol level, number of follicles, number of eggs retrieved) related to genotype were tested using a step-down bootstrap resampling method (the STEPBOOT option in PROC MULTTEST in SAS) [60]. This method adjusts for multiple comparisons while maintaining relatively good statistical power compared to other methods and is applicable to both normal and non-normally distributed data [60]. Outcome differences were first tested separately for each polymorphism, followed by comparing combinations of alleles for the two polymorphisms. The association between the distributions of patients across alleles for the two polymorphisms was tested by Fisher’s Exact Test. Multivariable model predicting each outcome was calculated using linear regression for estradiol level and negative-binomial regression for number of follicles and eggs retrieved. The latter method is an extension of Poisson regression. SAS v.9.2 (SAS Institute, Cary, NC) was used for all statistical analyses. Polymorphisms and were analyzed for Hardy-Weinberg equilibrium and linkage disequilibrium (LD) using PyPop: Python of Population Genomics software [61], CubeX [62] and SPSS (IBM, Armonk, NY). Unadjusted odds ratios for OHSS risk were determined using SAS v.9.2 and SPSS.

## Results

A total of 172 patients underwent IVF and were genotyped. The mean age of the patient population was 36.8 years old (Table 1). The majority of the population was Caucasian (57.0%) followed by Asian/Pacific Islander (15.7%), non-Hispanic, African American (11.1%) and Hispanic (2%). Because there were few Hispanic patients, for analysis we grouped these individuals with those for which no self-identified ethnic/ancestral information was available (16.3% of sample). Median levels of ovarian reserve markers TSH, day 3 follicle-stimulating hormone (FSH) and day 3 estradiol (E_2_) levels, were 1.8 IU/ml, 7.0 IU/L, and 40.8 pg/ml, respectively. On average, patient gonadotropin stimulation lasted 12 days. Mean E_2_ levels on the day of hCG administration was 1780 pg/ml and the median number of follicles and eggs retrieved was 10 and 9, respectively. Eighteen patients (10.5%) experienced moderate to severe OHSS (Table 1). The OHSS cases in our sample were similar to the 154 non-OHSS patients on age and most relevant clinical variables, with the exception of being more likely to be Black, less likely to be Asian. OHSS cases also had higher numbers of follicles, a higher mean estradiol level on day of hCG and more eggs retrieved (Table 1). These three clinical differences were expected based on the nature of OHSS.

**Table 1:** Demographic, clinical, and genetic characteristics of the study sample (N = 172)

	**Total**	**OHSS Cases**	**Controls**
*Mean age (SD), Years*	36.8 (4.1)	35.2 (2.9)	37 (4.2)
Race*	--	--	--
*White non-Hispanic*	98 (57.0)	12 (66.7)	86 (55.8)
*Black, non-Hispanic*	19 (11.1)	5 (27.8)	14 (9.1)
*Asian/Pacific Islander*	27 (15.7)	1 (5.6)	26 (16.9)
*Hispanic/Missing*	28 (16.3)	0 (0.0)	28 (18.2)
TSH (IU/mL)**	1.8 (1.2-2.5)	1.8 (1.5-2.1)	1.8 (1.2-2.6)
Day 3 FSH level (IU/L)**	7.0 (5.4-8.5)	6.3 (4.5-7.3)	7.0 (5.5-8.5)
Day 3 Estradiol level (pg/mL)**	40.8 (30.0-55.7)	41.6 (33.3-57.8)	39.0 (30.0-54.7)
Total number of follicles**	10.0 (6.0-15)	18.0 (13.0-24.0)	9.0 (5.0-12.0)
Total number of days stimulated **	12 (10–13)	12 (11–13)	12 (10–13)
Estradiol level day of hCG **	1780 (1136–2401)	2606 (1953–3436)	1735 (988–2254)
Total number of eggs retrieved **	9 (5–14)	18 (13–23)	8 (4–13)

* All categorical variables are presented as N (%).

** Presented as median (IQR) due to highly skewed distributions.

Recently, a novel, potentially inactivating, mutation was detected that resided within the *ins*LQ insertion (CTGCA > CG) in a patient with poor oocyte recovery following IVF [44]. This mutation would not have been identified using fragment analysis (the commonly employed method for detecting *ins*LQ). As a result, we analyzed *ins*LQ through direct sequencing of a 291 base-pair region of exon/intron 1. As shown in Table 2, relative allelic proportions for no-*ins*LQ/no-*ins*LQ, no-*ins*LQ/*ins*LQ and *ins*LQ/*ins*LQ were ~0.62, 0.34 and 0.035, respectively (n = 172). Another polymorphism, rs4073366 G > C, occurs ~142 bp downstream of *ins*LQ and was detected during sequencing. We attempted alternative methods (TaqMan^®^ probe, alternative primer pairs) to omit rs4073366 from our study of *ins*LQ, but these yielded unreliable results. Consequently, we included this single nucleotide polymorphism (SNP) in our analysis because of its potential impact on any apparent associations between *ins*LQ and COH outcome. For rs4073366, G/G, G/C and C/C variants occurred at relative proportions of ~0.72, 0.25 and 0.03, respectively in our patient population. Both variants were in Hardy-Weinberg equilibrium (HWE) and *ins*LQ and rs4073366 were in significant LD (D’ = 1.0, *P* < 0.0001) as determined by pair-wise LD estimation (Table 2) (49). Homozygosity or heterozygosity for *ins*LQ was associated with not being CG or CC for rs4073366 (Table 3, *P* = 0.012). In addition, we did not find any individuals who were homozygous for both *ins*LQ and rs4073366, nor did we identify any patients who had the *ins*LQ, rs4073366 “C” haplotype (data not shown).

**Table 2:** Allelic frequencies and linkage disequilibrium for the rs4539842 (insLQ) and rs4073366 polymorphisms

**Variant**	**Frequency (n = 172)**	**D’**	**r**^**2**^	**LD *P*-value**^**a**^
*rs4539842 (insLQ)*	--	--	--	--
*no-insLQ/no-insLQ*	0.622 (107)	--	--	--
*no-insLQ/insLQ*	0.340 (59)	--	--	--
*insLQ/insLQ*	0.035 (6)	--	--	--
		--	--	--
*rs4073366*	--	--	--	--
*GG*	0.721 (124)	--	--	--
*GC*	0.250 (43)	--	--	--
*CC*	0.029 (5)	--	--	--
*insLQ /rs4073366*	--	1.00	0.0474	<0.0001

^**a**^ Both variants were in Hardy-Weinberg equilibrium (HWE).

**Table 3:** Frequency of *ins*LQ and rs4073366 (N = 172)*

	**no-insLQ/no-insLQ**	**no-insLQ/ *ins*LQ**	***ins*LQ/*ins*LQ**
***GG***	68 (39.5%)	50 (29.1%)	6 (3.5%)
***CG***	34 (19.8%)	9 (5.2%)	0
***CC***	5 (2.9%)	0	0

*n (%), Fisher’s exact test for association between *ins*LQ and rs4073366: *P* = 0.012.

Of the 172 patients, up to 17 patients were excluded (depending on the clinical endpoint) from multivariable analysis because of missing data values related to being screened at outside centers that did not have complete ovarian reserve testing or they did not undergo follicular monitoring on day of hCG administration. Both age and ORT (day 3 FSH, TSH and E2 levels) have been prognostic clinical indicators of outcome to COH (52). As a result, regression models predicting estradiol level on day of HCG, number of follicles, and number of eggs retrieved included age, day 3 FSH level, day 3 estradiol levels, and the *ins*LQ and rs4073366 polymorphisms as covariates. In the multivariable models (Table 4), patient age was significantly associated with lower E2 levels on day of hCG (*P* = 0.03), fewer number of follicles (*P* < 0.0001) and a lower number of eggs retrieved (*P* = 0.0002). Increasing basal FSH level was associated with fewer follicles (*P* = 0.009) and eggs retrieved (*P* = 0.0002), but not E2 levels (*P* = 0.46) on day of hCG. All three main outcomes were positively correlated with each other (Spearman r, *P* < 0.0001) (data not shown). Finally, self-identified race/ethnicity was not significantly associated with any of the three main patient outcome measures (data not shown).

**Table 4:** Adjusted regression of demographic, clinical and genetic predictors of estradiol level on day of hCG, follicle count, and egg count*

**Factor**	**Estradiol Level on Day of hCG (N = 157)**^**a**^			**Number of Follicles (N = 155)**^**b**^			**Number of Eggs Retrieved (N = 160)**^**c**^		
	**Estimate**	**95% CI**	**P-Value**	**Estimate**	**95% CI**	**P-Value**	**Estimate**	**95% CI**	**P-Value**
*Age*	−48.2	−90.3, -6.1	0.03	0.939	0.918, 0.961	<0.0001	0.951	0.927, 0.977	0.0002
*Day 3 FSH level*	−10.3	−38.0, 17.3	0.46	0.976	0.958, 0.994	0.0097	0.953	0.930, 0.977	0.0002
*Day 3 Estradiol level*	−2.5	−7.7, 2.7	0.34	0.999	.997, 1.002	0.57	0.998	0.995, 1.000	0.08
*insLQ/insLQ, GG*	5.9	−818.3, 830.2	0.98	0.963	0.617, 1.503	0.32	0.647	0.380, 1.111	0.11
*no-insLQ/insLQ, GG*	−197.2	−538.9, 144.4	0.26	0.909	0.755, 1.096	0.87	0.952	0.772, 1.174	0.64
*no-insLQ/no-insLQ, CC*	−784.1	−1,682.0, 113.7	0.09	0.903	0.557, 1.63	0.68	0.665	0.372, 1.189	0.17
*no-insLQ/no-insLQ, CG*	163.8	−224.3, 551.9	0.41	1.210	0.985, 1.488	0.07	1.211	0.963, 1.523	0.10

* Estimates adjusted for all other variables presented.

^a^ Linear regression with estimates of change in mean estradiol levels (pg/ml).

^b^ Negative binomial regression with estimates of relative odds of number of follicles.

^c^ Negative binomial regression with estimates of relative odds of number of eggs retrieved.

None of the pairwise differences comparing estradiol levels, number of follicles, and number of eggs retrieved among the 3 *ins*LQ genotypes reached statistical significance (not shown). Similarly, results for rs4073366 genotype were also not significant for these three outcomes (not shown). We next examined the 6 non-zero combination genotypic groups and, again, found no significant differences for any of the outcomes when each mean was compared to the overall mean (Table 5). However, there was a non-significant trend for those individuals who did not carry *ins*LQ and were heterozygous (GC) for rs4073366 (i.e. no-*ins*LQ/no-*ins*LQ + CG) to have increased mean estradiol level on day of hCG compared to the mean across all other groups (*P* = 0.10). The *ins*LQ polymorphism (and rs4073366) was not associated with IVF protocol type, age, or basal FSH or E2 levels (data not shown).

**Table 5:** **Mean (SD) of estradiol level, number of follicles and number of eggs retrieved by *ins*LQ and rs4073366**^**±**^

	**Number of patients**	**Estradiol level**	**Number of follicles**	**Number of eggs retrieved**
*no-insLQ/no-insLQ + GG*	64-66	1819.5 (986.3)	9.8 (5.7)	9.3 (6.7)
*no-insLQ/ insLQ + GG*	48-49	1922.4 (1033.5)	10.9 (6.7)	11.4 (8.8)
*insLQ/insLQ + GG*	6	1946.0 (860.6)	10.3 (8.9)	6.7 (6.3)
*no-insLQ/no-insLQ + CG*	32-34	2156.1 (1009.4.0)*	13.5 (7.4)	13.3 (7.9)
*no-insLQ/ insLQ + CG*	8-9	1232.3 (700.3)	7.8 (3.7)	9.4 (3.7)
*no-insLQ/no-insLQ + CC*	5	1201.6 (464.9)	10.4 (7.4)	7.2 (0.83)

* *P* = 0.10.

The *ins*LQ variant is purportedly a high-function allele and, accordingly, might place patients at a higher risk of OHSS. As a result, we tested whether either *ins*LQ or rs4073366 were associated with an increased risk of OHSS. Because of the small number of OHSS patients, logistic regression analyses were performed without adjustment for covariates. Intriguingly, the *ins*LQ variant was not associated with OHSS by either genotype (*P* = 0.788) (data not shown) or *ins*LQ carrier status (Table 6). There was a non-significant (P = 0.07) trend towards an association between rs4073366 genotype and OHSS. When rs4073366 carrier status was included in our analysis, carriers of the rs4073366 C allele exhibited a significantly (P = 0.033) higher risk of OHSS (OR = 2.95, 95% CI = 1.09-7.96). Further haplotype analysis revealed that only the no-*ins*LQ/C haplotype was significant (*P* = 0.023) for OHSS risk (OR = 2.46, 95% CI 1.11-5.46) (data not shown).

**Table 6:** **Analysis of *ins*LQ/ rs4539842 and rs4073366 carrier status and OHSS risk**

**Variant**	**OHSS Cases (%)**	**Controls (%)**	**OR (95% CI)**^**#**^	P-value*
***ins*LQ/rs4073366**				
Non-Carrier	12 (66.7)	95 (61.7)		
Carrier	6 (33.3)	59 (38.3)	0.81 (0.29-2.26)	0.6807
**rs4539842 (C Allele)**				
Non-carrier	9 (50.0)	115 (74.7)		
Carrier	9 (50.0)	39 (25.3)	2.95 (1.09-7.96)	0.0328

^#^ Unadjusted OR.

^*^ Logistic regression.

## Discussion

LHR-mediated signaling plays an important role in patient response to exogenous gonadotropins (i.e. hCG) administered during COH and inter-individual variability in LHR activity could significantly impact outcome. As a result, we investigated whether *LHCGR* genetic variation influences response to COH. We focused our analysis on the *ins*LQ polymorphism (rs4539842) and the rs4073366 G > C SNP located downstream of *ins*LQ in intron 1. We found that insLQ was not associated with patient response to COH, nor was it a predictor for OHSS. Therefore, it is possible that the improved function conferred to LHR by this polymorphism *in vitro*[52] is not reflective of the situation in the ovaries. Moreover, *ins*LQ activity was previously investigated in HEK-293 cells which may not accurately replicate the behavior of the *ins*LQ receptor variant ovarian granulosa cells [52]. In contrast, we found that carriers of the rs4073366 “C” allele exhibited a ~3-fold increased risk of developing OHSS.

Very little information exists regarding polymorphisms that are predictive of patients developing OHSS. For example, the *FSHR* Thr307Ala polymorphism has been linked with iatrogenic OHSS in an Indian population [63], but not in European [9] or Brazilian patients [64]. The well-studied Asn680Ser *FSHR* polymorphism has not been associated with OHSS, but it is potentially predictive of the severity of symptoms [9]. In addition, *BMP15* variation, such as the rs3810682 SNP (OR = 2.7, 95% CI = 1.3-5.7), has also been implicated in OHSS [23,65]. Finally, *VEGFA* gene variation was recently identified as a risk allele (OR = 3.4, 95% CI = 1.01-11.7) for OHSS [66]. Given the paucity of genetic risk factors for OHSS, one of the most significant findings of this work was the association of rs4073366 C allele carrier status with increased risk of OHSS (OR = 2.95, 95% CI 1.09-7.96, Table 6) during COH. Furthermore, it seems that the effect of rs4073366 on OHSS risk was largely related to the no-insLQ/C haplotype (OR = 2.46, 95% CI 1.11-5.46) (data not shown). This interesting finding requires further investigation in other COH populations and suggests that the LHCGR genetic variation influences OHSS development. A recent genome-wide association study found that *LHCGR* was associated with serum steroid hormone binding globulin (SHBGs) levels (62), which could result in elevated serum concentrations of androgens and estrogen. However, the impact of LHCGR polymorphisms (i.e. *ins*LQ, rs4073366) on SHBG levels in not currently known.

While rs4073366 is a potential predictor of OHSS risk, the functional consequences of this polymorphism on LHR function are yet to be elucidated. rs4073366 has a major allele of “C” on the “+” strand (“G” on the “-“strand in this study) and resides in a cryptic 3’ splice acceptor site (data not shown) which could potentially impact *LHCGR* mRNA processing yielding a splice variant with altered activity [67]. In addition, the intronic region surrounding rs4073366 is complementary to *APOE* mRNA and has been associated with decreased risk of Alzheimer’s disease (AD) in males carrying the *APOE* ϵ4 allele [67]. Given that apolipoprotein E is important for cholesterol uptake and steroidogenesis, and genetic variation in *APOE* has been linked to reproductive efficiency [68-70], it is possible that rs4073366 may alter response to fertility drugs via modulation of *APOE* mRNA stability. Although beyond the scope of this investigation; future work is focused on investigating the molecular consequences of rs4073366 on LHCGR function.

## Conclusions

Outcome to COH is multi-factorial, variable and unpredictable. There have been few studies that have investigated *LHCGR* variability and its influence on COH. Here, we provide the first report of an association between *LHCGR* genetic variability and OHSS risk. The relevance of the rs4073366 polymorphism OHSS should be evaluated in additional patient populations. In addition, because OHSS is multigenic in nature, future work is warranted to investigate whether rs4073366, and other *LHCGR* variants, genetically interact with other loci to predict patient response to COH.

## Abbreviations

COH: Controlled ovarian hyperstimulation; OHSS: Ovarian hyperstimulation syndrome; SNP: Single nucleotide polymorphism.

## Competing interests

The authors declare that they have no competing interests associated with this work.

## Authors’ contributions

TO: Conceived of the study, participated in its design, carried out molecular analyses, assisted in data analysis and drafted the manuscript. MK: Conducted statistical analyses, assisted with drafting the manuscript. AH: Participated in study design, assisted in data analysis and drafted the manuscript. AC: Carried out molecular analysis with TO. IG: Processed samples, carried out molecular analysis with TO. SS: Worked with MK on all statistical analyses. DF: Conceived of the study, participated in its design, recruited participants, assisted with drafting the manuscript. PG: Conceived of the study, participated in its design, recruited participants, assisted with drafting the manuscript. All authors read and approved the final manuscript.

## References

[B1] Scott RT Toner JP Muasher SJ Oehninger S Robinson S Rosenwaks Z Follicle-stimulating hormone levels on cycle day 3 are predictive of in vitro fertilization outcome Fertil Steril 1989 51 651 654 2494082 10.1016/s0015-0282(16)60615-5

[B2] Licciardi FL Liu HC Rosenwaks Z Day 3 estradiol serum concentrations as prognosticators of ovarian stimulation response and pregnancy outcome in patients undergoing in vitro fertilization Fertil Steril 1995 64 991 994 7589648 10.1016/s0015-0282(16)57916-3

[B3] Ficicioglu C Kutlu T Baglam E Bakacak Z Early follicular antimullerian hormone as an indicator of ovarian reserve Fertil Steril 2006 85 592 596 10.1016/j.fertnstert.2005.09.019 16500324

[B4] Hsu A Arny M Knee AB Bell C Cook E Novak AL Grow DR Antral follicle count in clinical practice: analyzing clinical relevance Fertil Steril 2011 95 474 479 10.1016/j.fertnstert.2010.03.023 20434151

[B5] Alviggi C Humaidan P Ezcurra D Hormonal, functional and genetic biomarkers in controlled ovarian stimulation: tools for matching patients and protocols Reprod Biol Endocrinol 2012 10 9 10.1186/1477-7827-10-9 22309877 PMC3299595

[B6] Altmae S Hovatta O Stavreus-Evers A Salumets A Genetic predictors of controlled ovarian hyperstimulation: where do we stand today? Hum Reprod Update 2011 17 813 828 10.1093/humupd/dmr034 21862569

[B7] Anagnostou E Mavrogianni D Theofanakis C Drakakis P Bletsa R Demirol A Gurgan T Antsaklis A Loutradis D ESR1, ESR2 and FSH receptor gene polymorphisms in combination: a useful genetic tool for the prediction of poor responders Curr Pharm Biotechnol 2012 13 426 434 10.2174/138920112799361891 21658000

[B8] Boudjenah R Molina-Gomes D Torre A Bergere M Bailly M Boitrelle F Taieb S Wainer R Benahmed M De Mazancourt P Genetic polymorphisms influence the ovarian response to rFSH stimulation in patients undergoing in vitro fertilization programs with ICSI PLoS One 2012 7 e38700 10.1371/journal.pone.0038700 22701696 PMC3372493

[B9] Daelemans C Smits G De Maertelaer V Costagliola S Englert Y Vassart G Delbaere A Prediction of severity of symptoms in iatrogenic ovarian hyperstimulation syndrome by follicle-stimulating hormone receptor Ser680Asn polymorphism J Clin Endocrinol Metab 2004 89 6310 6315 10.1210/jc.2004-1044 15579795

[B10] De Castro F Moron FJ Montoro L Galan JJ Hernandez DP Padilla ES Ramirez-Lorca R Real LM Ruiz A Human controlled ovarian hyperstimulation outcome is a polygenic trait Pharmacogenetics 2004 14 285 293 10.1097/00008571-200405000-00003 15115914

[B11] Desai SS Achrekar SK Pathak BR Desai SK Mangoli VS Mangoli RV Mahale SD Follicle-stimulating hormone receptor polymorphism (G-29A) is associated with altered level of receptor expression in Granulosa cells J Clin Endocrinol Metab 2011 96 2805 2812 10.1210/jc.2011-1064 21752882

[B12] Jun JK Yoon JS Ku SY Choi YM Hwang KR Park SY Lee GH Lee WD Kim SH Kim JG Moon SY Follicle-stimulating hormone receptor gene polymorphism and ovarian responses to controlled ovarian hyperstimulation for IVF-ET J Hum Genet 2006 51 665 670 10.1007/s10038-006-0005-5 16871362

[B13] Loutradis D Patsoula E Minas V Koussidis GA Antsaklis A Michalas S Makrigiannakis A FSH receptor gene polymorphisms have a role for different ovarian response to stimulation in patients entering IVF/ICSI-ET programs J Assist Reprod Genet 2006 23 177 184 10.1007/s10815-005-9015-z 16758348 PMC3454958

[B14] Lussiana C Guani B Mari C Restagno G Massobrio M Revelli A Mutations and polymorphisms of the FSH receptor (FSHR) gene: clinical implications in female fecundity and molecular biology of FSHR protein and gene Obstet Gynecol Surv 2008 63 785 795 10.1097/OGX.0b013e31818957eb 19017414

[B15] Sheikhha MH Eftekhar M Kalantar SM Investigating the association between polymorphism of follicle-stimulating hormone receptor gene and ovarian response in controlled ovarian hyperstimulation J Hum Reprod Sci 2011 4 86 90 10.4103/0974-1208.86089 22064672 PMC3205539

[B16] Wunsch A Ahda Y Banaz-Yasar F Sonntag B Nieschlag E Simoni M Gromoll J Single-nucleotide polymorphisms in the promoter region influence the expression of the human follicle-stimulating hormone receptor Fertil Steril 2005 84 446 453 10.1016/j.fertnstert.2005.02.031 16084888

[B17] Wunsch A Sonntag B Simoni M Polymorphism of the FSH receptor and ovarian response to FSH Ann Endocrinol (Paris) 2007 68 160 166 10.1016/j.ando.2007.04.006 17544358

[B18] Altmae S Haller K Peters M Hovatta O Stavreus-Evers A Karro H Metspalu A Salumets A Allelic estrogen receptor 1 (ESR1) gene variants predict the outcome of ovarian stimulation in in vitro fertilization Mol Hum Reprod 2007 13 521 526 10.1093/molehr/gam035 17540666

[B19] Georgiou I Konstantelli M Syrrou M Messinis IE Lolis DE Oestrogen receptor gene polymorphisms and ovarian stimulation for in-vitro fertilization Hum Reprod 1997 12 1430 1433 10.1093/humrep/12.7.1430 9262271

[B20] Loutradis D Theofanakis C Anagnostou E Mavrogianni D Partsinevelos GA Genetic profile of SNP(s) and ovulation induction Curr Pharm Biotechnol 2012 13 417 425 10.2174/138920112799361954 21657995

[B21] Altmae S Haller K Peters M Saare M Hovatta O Stavreus-Evers A Velthut A Karro H Metspalu A Salumets A Aromatase gene (CYP19A1) variants, female infertility and ovarian stimulation outcome: a preliminary report Reprod Biomed Online 2009 18 651 657 10.1016/S1472-6483(10)60009-0 19549443

[B22] Wang TT Wu YT Dong MY Sheng JZ Leung PC Huang HF G546A polymorphism of growth differentiation factor-9 contributes to the poor outcome of ovarian stimulation in women with diminished ovarian reserve Fertil Steril 2010 94 2490 2492 10.1016/j.fertnstert.2010.03.070 20451184

[B23] Hanevik HI Hilmarsen HT Skjelbred CF Tanbo T Kahn JA A single nucleotide polymorphism in BMP15 is associated with high response to ovarian stimulation Reprod Biomed Online 2011 23 97 104 10.1016/j.rbmo.2011.02.015 21565556

[B24] Hillier SG Gonadotropic control of ovarian follicular growth and development Mol Cell Endocrinol 2001 179 39 46 10.1016/S0303-7207(01)00469-5 11420129

[B25] Webb R Nicholas B Gong JG Campbell BK Gutierrez CG Garverick HA Armstrong DG Mechanisms regulating follicular development and selection of the dominant follicle Reprod Suppl 2003 61 71 90 14635928

[B26] Ziecik AJ Kaczmarek MM Blitek A Kowalczyk AE Li X Rahman NA Novel biological and possible applicable roles of LH/hCG receptor Mol Cell Endocrinol 2007 269 51 60 10.1016/j.mce.2006.08.016 17367919

[B27] Ziecik AJ Derecka-Reszka K Rzucidlo SJ Extragonadal gonadotropin receptors, their distribution and function J Physiol Pharmacol 1992 43 33 49 1343973

[B28] Ziecik AJ Derecka K Gawronska B Stepien A Bodek G Nongonadal LH/hCG receptors in pig: functional importance and parallels to human Semin Reprod Med 2001 19 19 30 10.1055/s-2001-13907 11394200

[B29] Gawronska B Paukku T Huhtaniemi I Wasowicz G Ziecik AJ Oestrogen-dependent expression of LH/hCG receptors in pig Fallopian tube and their role in relaxation of the oviduct J Reprod Fertil 1999 115 293 301 10.1530/jrf.0.1150293 10434935

[B30] Perrier d’Hauterive S Berndt S Tsampalas M Charlet-Renard C Dubois M Bourgain C Hazout A Foidart JM Geenen V Dialogue between blastocyst hCG and endometrial LH/hCG receptor: which role in implantation? Gynecol Obstet Invest 2007 64 156 160 10.1159/000101740 17934312

[B31] Hearn MT Gomme PT Molecular architecture and biorecognition processes of the cystine knot protein superfamily: part I. The glycoprotein hormones J Mol Recognit 2000 13 223 278 10.1002/1099-1352(200009/10)13:5<223::AID-JMR501>3.0.CO;2-L 10992290

[B32] Ascoli M Fanelli F Segaloff DL The lutropin/choriogonadotropin receptor, a 2002 perspective Endocr Rev 2002 23 141 174 10.1210/er.23.2.141 11943741

[B33] Kumar P Sait SF Sharma A Kumar M Ovarian hyperstimulation syndrome J Hum Reprod Sci 2011 4 70 75 10.4103/0974-1208.86080 22065820 PMC3205536

[B34] Cerrillo M Pacheco A Rodriguez S Gomez R Delgado F Pellicer A Garcia-Velasco JA Effect of GnRH agonist and hCG treatment on VEGF, angiopoietin-2, and VE-cadherin: trying to explain the link to ovarian hyperstimulation syndrome Fertil Steril 2011 95 2517 2519 10.1016/j.fertnstert.2010.12.054 21269615

[B35] Vardhana PA Julius MA Pollak SV Lustbader EG Trousdale RK Lustbader JW A unique human chorionic gonadotropin antagonist suppresses ovarian hyperstimulation syndrome in rats Endocrinology 2009 150 3807 3814 10.1210/en.2009-0107 19443574 PMC2717881

[B36] Van de Lagemaat R Raafs BC Van Koppen C Timmers CM Mulders SM Hanssen RG Prevention of the onset of ovarian hyperstimulation syndrome (OHSS) in the rat after ovulation induction with a low molecular weight agonist of the LH receptor compared with hCG and rec-LH Endocrinology 2011 152 4350 4357 10.1210/en.2011-1077 21896671

[B37] Fanelli F Puett D Structural aspects of luteinizing hormone receptor: information from molecular modeling and mutagenesis Endocrine 2002 18 285 293 10.1385/ENDO:18:3:285 12450321

[B38] Fanelli F Themmen AP Puett D Lutropin receptor function: insights from natural, engineered, and computer-simulated mutations IUBMB Life 2001 51 149 155 10.1080/152165401753544214 11547916

[B39] Fanelli F Verhoef-Post M Timmerman M Zeilemaker A Martens JW Themmen AP Insight into mutation-induced activation of the luteinizing hormone receptor: molecular simulations predict the functional behavior of engineered mutants at M398 Mol Endocrinol 2004 18 1499 1508 10.1210/me.2003-0050 15016840

[B40] Themmen AP An update of the pathophysiology of human gonadotrophin subunit and receptor gene mutations and polymorphisms Reprod Suppl 2005 130 263 274 10.1530/rep.1.00663 16123233

[B41] Minegishi T Nakamura K Takakura Y Miyamoto K Hasegawa Y Ibuki Y Igarashi M Minegish T Cloning and sequencing of human LH/hCG receptor cDNA Biochem Biophys Res Commun 1990 172 1049 1054 10.1016/0006-291X(90)91552-4 2244890

[B42] Atger M Misrahi M Sar S Le Flem L Dessen P Milgrom E Structure of the human luteinizing hormone-choriogonadotropin receptor gene: unusual promoter and 5’ non-coding regions Mol Cell Endocrinol 1995 111 113 123 10.1016/0303-7207(95)03557-N 7556872

[B43] Mongan NP Hughes IA Lim HN Evidence that luteinising hormone receptor polymorphisms may contribute to male undermasculinisation Eur J Endocrinol 2002 147 103 107 10.1530/eje.0.1470103 12088926

[B44] Bentov Y Kenigsberg S Casper RF A novel luteinizing hormone/chorionic gonadotropin receptor mutation associated with amenorrhea, low oocyte yield, and recurrent pregnancy loss Fertil Steril 2012 97 1165 1168 10.1016/j.fertnstert.2012.02.002 22369774

[B45] Simoni M Tuttelmann F Michel C Bockenfeld Y Nieschlag E Gromoll J Polymorphisms of the luteinizing hormone/chorionic gonadotropin receptor gene: association with maldescended testes and male infertility Pharmacogenet Genomics 2008 18 193 200 10.1097/FPC.0b013e3282f4e98c 18300940

[B46] Kossack N Troppmann B Richter-Unruh A Kleinau G Gromoll J Aberrant transcription of the LHCGR gene caused by a mutation in exon 6A leads to Leydig cell hypoplasia type II Mol Cell Endocrinol 2013 366 59 67 10.1016/j.mce.2012.11.018 23232123

[B47] Latronico AC Arnhold IJ Inactivating mutations of the human luteinizing hormone receptor in both sexes Semin Reprod Med 2012 30 382 386 10.1055/s-0032-1324721 23044874

[B48] Yariz KO Walsh T Uzak A Spiliopoulos M Duman D Onalan G King MC Tekin M Inherited mutation of the luteinizing hormone/choriogonadotropin receptor (LHCGR) in empty follicle syndrome Fertil Steril 2011 96 e125 130 10.1016/j.fertnstert.2011.05.057 21683950 PMC3143235

[B49] Qiao J Han B Liu BL Chen X Ru Y Cheng KX Chen FG Zhao SX Liang J Lu YL A splice site mutation combined with a novel missense mutation of LHCGR cause male pseudohermaphroditism Hum Mutat 2009 30 E855 865 10.1002/humu.21072 19551906

[B50] Arnhold IJ Lofrano-Porto A Latronico AC Inactivating mutations of luteinizing hormone beta-subunit or luteinizing hormone receptor cause oligo-amenorrhea and infertility in women Horm Res 2009 71 75 82 19129711 10.1159/000183895

[B51] Segaloff DL Diseases associated with mutations of the human lutropin receptor Prog Mol Biol Transl Sci 2009 89 97 114 20374734 10.1016/S1877-1173(09)89004-2

[B52] Piersma D Berns EM Verhoef-Post M Uitterlinden AG Braakman I Pols HA Themmen AP A common polymorphism renders the luteinizing hormone receptor protein more active by improving signal peptide function and predicts adverse outcome in breast cancer patients J Clin Endocrinol Metab 2006 91 1470 1476 10.1210/jc.2005-2156 16464948

[B53] Piersma D Themmen AP Look MP Klijn JG Foekens JA Uitterlinden AG Pols HA Berns EM GnRH and LHR gene variants predict adverse outcome in premenopausal breast cancer patients Breast Cancer Res 2007 9 R51 10.1186/bcr1756 17692113 PMC2206727

[B54] Rodien P Cetani F Costagliola S Tonacchera M Duprez L Minegishi T Govaerts C Vassart G Evidences for an allelic variant of the human LC/CG receptor rather than a gene duplication: functional comparison of wild-type and variant receptors J Clin Endocrinol Metab 1998 83 4431 4434 10.1210/jc.83.12.4431 9851790

[B55] Tsai-Morris CH Geng Y Buczko E Dehejia A Dufau ML Genomic distribution and gonadal mRNA expression of two human luteinizing hormone receptor exon 1 sequences in random populations Hum Hered 1999 49 48 51 10.1159/000022840 9858858

[B56] Chen ZJ Zhao H He L Shi Y Qin Y Shi Y Li Z You L Zhao J Liu J Genome-wide association study identifies susceptibility loci for polycystic ovary syndrome on chromosome 2p16.3, 2p21 and 9q33.3 Nat Genet 2011 43 55 59 10.1038/ng.732 21151128

[B57] Kerkela E Skottman H Friden B Bjuresten K Kere J Hovatta O Exclusion of coding-region mutations in luteinizing hormone and follicle-stimulating hormone receptor genes as the cause of ovarian hyperstimulation syndrome Fertil Steril 2007 87 603 606 10.1016/j.fertnstert.2006.06.060 17074323

[B58] Bergh PA Navot D Ovarian hyperstimulation syndrome: a review of pathophysiology J Assist Reprod Genet 1992 9 429 438 10.1007/BF01204048 1482837

[B59] Navot D Bergh PA Laufer N Ovarian hyperstimulation syndrome in novel reproductive technologies: prevention and treatment Fertil Steril 1992 58 249 261 1633889 10.1016/s0015-0282(16)55188-7

[B60] Westfall PH, Wolfinger RD Closed multiple testing procedures and PROC MULTTEST 2000 Estados Unidos: Observations SAS Institute Inc.

[B61] Lancaster AK Single RM Solberg OD Nelson MP Thomson G PyPop update–a software pipeline for large-scale multilocus population genomics Tissue Antigens 2007 69 Suppl 1 192 197 17445199 10.1111/j.1399-0039.2006.00769.x PMC4369784

[B62] Gaunt TR Rodriguez S Day IN Cubic exact solutions for the estimation of pairwise haplotype frequencies: implications for linkage disequilibrium analyses and a web tool ‘CubeX’ BMC Bioinformatics 2007 8 428 10.1186/1471-2105-8-428 17980034 PMC2180187

[B63] Achrekar SK Modi DN Desai SK Mangoli VS Mangoli RV Mahale SD Follicle-stimulating hormone receptor polymorphism (Thr307Ala) is associated with variable ovarian response and ovarian hyperstimulation syndrome in Indian women Fertil Steril 2009 91 432 439 10.1016/j.fertnstert.2007.11.093 18321487

[B64] d’Alva CB Serafini P Motta E Kohek MB Latronico AC Mendonca BB Absence of follicle-stimulating hormone receptor activating mutations in women with iatrogenic ovarian hyperstimulation syndrome Fertil Steril 2005 83 1695 1699 10.1016/j.fertnstert.2004.12.044 15950638

[B65] Moron FJ De Castro F Royo JL Montoro L Mira E Saez ME Real LM Gonzalez A Manes S Ruiz A Bone morphogenetic protein 15 (BMP15) alleles predict over-response to recombinant follicle stimulation hormone and iatrogenic ovarian hyperstimulation syndrome (OHSS) Pharmacogenet Genomics 2006 16 485 495 10.1097/01.fpc.0000215073.44589.96 16788381

[B66] Hanevik HI Hilmarsen HT Skjelbred CF Tanbo T Kahn JA Increased risk of ovarian hyperstimulation syndrome following controlled ovarian hyperstimulation in patients with vascular endothelial growth factor +405 cc genotype Gynecol Endocrinol 2012 28 845 849 10.3109/09513590.2012.683056 22587628

[B67] Haasl RJ Ahmadi MR Meethal SV Gleason CE Johnson SC Asthana S Bowen RL Atwood CS A luteinizing hormone receptor intronic variant is significantly associated with decreased risk of Alzheimer’s disease in males carrying an apolipoprotein E epsilon4 allele BMC Med Genet 2008 9 37 18439297 10.1186/1471-2350-9-37 PMC2396156

[B68] Corbo RM Ulizzi L Scacchi R Martinez-Labarga C De Stefano GF Apolipoprotein E polymorphism and fertility: a study in pre-industrial populations Mol Hum Reprod 2004 10 617 620 10.1093/molehr/gah082 15220465

[B69] Corbo RM Ulizzi L Piombo L Scacchi R Study on a possible effect of four longevity candidate genes (ACE, PON1, PPAR-gamma, and APOE) on human fertility Biogerontology 2008 9 317 323 10.1007/s10522-008-9143-9 18443916

[B70] Corbo RM Scacchi R Cresta M Differential reproductive efficiency associated with common apolipoprotein e alleles in postreproductive-aged subjects Fertil Steril 2004 81 104 107 10.1016/j.fertnstert.2003.05.029 14711551

